# Travelling with(in) critical AI studies: An east Asian standpoint

**DOI:** 10.1177/01634437251385483

**Published:** 2025-10-21

**Authors:** Hiu-Fung Chung

**Affiliations:** 1 University of Toronto, ON, Canada

**Keywords:** critical AI studies, east Asia, Asian media studies, traveling theory, global standpoints

## Abstract

How does critical AI studies itself travel, and what happens when it arrives elsewhere? This commentary reflects on the global circulation of critique from the standpoint of East Asia, where layered histories of modernization, technological aspiration, and cultural traditions generate alternative ways of knowing, imagining, and critiquing AI beyond South–North frameworks. Writing from my position as a graduate student from East Asia, now trained in a North American institution, I introduce three analytical vectors—*tangle, transplant*, and *transmute*—all of which emerge dialogically through my research on AI innovation in East Asia and through engagement with Asian media scholarship addressing similar concerns. Through vignettes from this research journey, I suggest that these themes illuminate how dominant critical vocabularies encounter local imaginaries, on-the-ground frictions, socio-cultural histories, and divergent ethical orientations. Rather than proposing a unified Asian critical theory of AI, I offer “traveling AI” as a reflexive praxis that centers relational co-constitution, situated reworking, and philosophical reorientation, while remaining attuned to epistemic tensions and power differentials. In dialogue with broader de-westernizing projects, this paper suggests that East Asia can contribute to reimagining critique not as theory from the center or the periphery, but as an *ongoing praxis of troubling with in-betweenness*.

## Traveling with troubles: reworking critical AI from elsewhere

Academic life is full of travel and trouble—across borders, between disciplines, among concepts, and within methods. When I arrived in Canada in late 2022 to begin my doctoral studies in media, technology, and culture, transnational mobility was still shaped by the lingering effects of the COVID-19 pandemic. But it is not only scholars who travel. Artificial intelligence (AI) systems, along with their imaginaries, operations, and critiques, also circulate across borders as assemblages of knowledge, capital, labor, and power. The viral spread of tools like ChatGPT, Copilot, and Perplexity has amplified the global visibility of generative AI, bringing renewed momentum to “critical AI studies,” an emerging interdisciplinary field informed by traditions in critical algorithm studies, critical data studies, and other diverse theoretical trajectories.

Yet as I tried to situate my own work, I found myself a graduate student trained in East Asia and now studying in North America, facing another kind of trouble: how to think and work critically with AI from East Asia without either reproducing Eurocentric logics or uncritically adopting the vocabularies of Global South critiques. While Global South approaches are insightful for challenging universalist assumptions and resisting the “tropicalization” of knowledge under Northern epistemologies (Grohmann, 2025; Soares Seto, 2025), they often leave unresolved how to meaningfully engage with regions such as East Asia, which do not fit neatly into South–North spatial and ideological binaries. Alternative paradigms like the “Global East” (see Müller, 2020) offer important reorientations by articulating the East as a liminal epistemic space, but these efforts often remain overly China-centric (Cai, 2025; Zeng et al., 2022), due not only to geopolitical, technological, and economic hierarchies, but also to structural inequalities in global academic visibility.

How, then, might we attend to the heterogeneous AI imaginaries, innovations, and implementations across East Asia, where societies are embedded in multiple temporalities, cultural traditions, and sociotechnical conditions, yet do not fit neatly within South-North or East-West frameworks?

In this short essay, I draw upon and extend these situated dialogues in *Media, Culture & Society*, exemplified by the special issue “Encounters with Western Media Theory,” which foregrounds the epistemic struggles of academic knowledge production and the redefinition of research, citational, and pedagogical practices in the globalized field of media and communication studies (Keightley et al., 2023). Specifically, I reflect on how critical AI studies—its concepts and theoretical assumptions—travel and transform through encounters with East Asian contexts. My reflections are grounded in my positionality as a media sociologist moving between Asian and North American academic institutions, asking what kinds of frictions, translations, and conceptual shifts unfold when critique is put in motion. To speak of an East Asian standpoint is not to claim regional coherence or theoretical unity. Rather, it is to underscore the generative tensions that arise from postcolonial legacies, uneven trajectories of development, and diverse philosophies of technology, all of which, I argue, offer potentialities for reframing and reworking the epistemic foundations of critical AI studies.

## Traveling AI: a critical standpoint with east Asian media studies

Critical AI studies, despite its interdisciplinary vibrancy and timeliness, contends with an analytical challenge: *how to engage with the global movements of the field without flattening the situatedness and vitality of its interventions*. In a broad sense, the emerging field interrogates the social, political, economic, and epistemological conditions under which artificial intelligence systems are imagined, designed, and deployed, as well as the operations of power and inequality configuring them (Grohmann, 2025; Lindgren, 2023). Its contributions range from critiques of AI-amplified surveillance capitalism (Nagy, 2024; Zuboff, 2022) to investigations of automated inequality and technocratic governance (Eubanks, 2018; Zajko, 2023). Yet regardless of its global claims—both empirical and theoretical, critical AI studies remains largely anchored in Western theoretical traditions, including the Frankfurt School, Anglo-European political theory (Rafanelli, 2022; Tacheva and Ramasubramanian, 2023), and Northern science and technology studies (Roberge et al., 2020).

These perspectives, while influential and insightful, risk reproducing a long-standing geopolitical asymmetry, both discursively and materially, that positions the Global North as the primary source of theoretical inspiration while casting the Global South or East as sites of application, extension, or reconstruction. Recent interventions, in line with various de-westernizing, decolonial, and Indigenous intellectual projects, have challenged this meta-theoretical imbalance. Grohmann (2025), for instance, calls for recognizing Latin America not merely as a “field site” but as a living space of epistemological and methodological contribution to global debates in critical data studies. Natale et al. (2025) remind us that although AI is both locally embedded and global in scope, the majority of critical scholarship often emphasizes local contexts of AI without adequately problematizing or situating them within global power geometries to show how actors, institutions, and epistemologies interact across uneven terrains. In my home discipline of media and communication studies, related interventions have gained traction. Inspired by feminist standpoint theory (e.g. Harding, 1991), Girginova et al. (2025), for example, advocate for a global communication standpoint as a generative epistemology that is contextually grounded, historically informed, attentive to power, and committed to comparison through situated difference. These efforts open up space to reimagine more reciprocal and plural forms of critical engagement with the global–local dynamics of AI.

It is within this context that I situate my reflection on what it means to articulate a critical standpoint on AI from East Asia. I approach East Asia strategically, as an *analytic vantage point* rather than a bounded geographical location, in order to move beyond the pigeonholing of scholarship (Cheruiyot and Ferrer-Conill, 2021) that relegates non-Western contexts to a purely descriptive or subordinate role in Northern-centric critical AI studies. Crucially, this strategic positioning does not endorse a distinctly “Asian” version of epistemic fundamentalism (Nikoi, 2019), nor does it reproduce the Orientalist terminologies that underpin dominant media and communication scholarship (Ranji, 2021).

Against this backdrop, I introduce three conceptual vectors, namely *tangle, transplant*, and *transmute*, as an alternative way of thinking *with* and *from* East Asia without collapsing it into a unified regional theory. Drawing on the idea of traveling theory (Bal, 2009; Said, 2000), I examine how concepts and approaches within critical AI studies, when they cross geographical borders and historical moments, are refracted, resisted, and remade through frictions between global abstractions and situated realities. Following feminist anthropologist Tsing (2005), I understand friction not as simple resistance but as the unequal, unstable, and generative qualities of interconnection across differences that allow conceptual transformation to occur. To further sustain this reflexive orientation, I also draw on what Said (1978, cited in Ranji, 2021) calls “methodological self-consciousness,” a continual self-examination of methodology and theorizing practice that attends both to the researcher’s positioning in relation to the empirics and to the discursive formations that confer authority, ensuring critique remains responsive to lived realities rather than doctrinal preconceptions. Informed by this epistemic standpoint, the “3T” framework is grounded in my own research travels on AI innovation in East Asia and informed by Asian media scholars who have likewise grappled with the tensions of theorizing from, and in relation to, the region and beyond.

### Tangle: relational co-constitution and variegated AI imaginaries

Innovation is always about how the future is imagined, narrated, and materialized as desirable in specific institutional, discursive, and political settings. This concern resonates with the concept of sociotechnical imaginaries, a well-established area of inquiry in science and technology studies (STS). The notion of tangle surfaced during the early phase of my research project, as I followed how AI futures were being narrated and legitimized across East Asia. While prior research has compared China’s national AI strategy with those of the United States and Europe (Bareis and Katzenbach, 2022) and explored Japan’s specific technocultural trajectory in digitization and robotics (Robertson, 2018; Spremberg, 2024), I am interested in how other Asian authorities envision AI-driven futures. I turned to three cases, including Hong Kong, Taiwan, and Singapore, that share comparable developmental trajectories but differ in political regime. Although Singapore is not geographically part of East Asia, it is frequently grouped with Taiwan, Hong Kong, and South Korea in comparative analyses of socio-economic development, political change, and welfare policy (Peng and Wong, 2008), particularly through the enduring legacy of the “Four Asian Tigers.” This framing continues to shape how these societies are positioned in regional debates on technocratic governance, economic modernization, and competitive capitalism.

At first glance, the imaginaries of AI in Hong Kong, Taiwan, and Singapore appeared distinct and internally coherent. In Hong Kong, AI was depicted as a post-crisis strategy to re-enter regional innovation races. Taiwan, building on its legacy in semiconductor manufacturing, positioned AI within a broader project of digital democracy and economic nationalism. Singapore, in contrast, casted AI development through the language of administrative optimization and long-term planning under its Smart Nation initiative. However, reading across these cases revealed that they were not simply parallel or independent trajectories. Instead, they were informed by a shared regional condition shaped by colonial modernities, geopolitics, and aspirations for developmental competitiveness. What came into view through this comparison was a techno-developmental imaginary that treats AI as both inevitable and essential. In all three cases, AI was understood as a strategic resource to strengthen computational capacity and manage uncertainty amid intensifying global competition (Chung, 2025).

Despite this convergence, the meanings and performative roles of AI diverge in critical ways across institutional, technological, and sociopolitical contexts. Singapore promotes a cybernetic pragmatism rooted in technocratic governance. Taiwan positions AI as both a democratic shield and an economic buffer in response to geopolitical risk, especially in the context of US–China rivalry. Hong Kong embeds AI within entrepreneurial intermediation shaped by the looming presence of Chinese state capitalism. These imaginaries cannot be reduced to narratives of national exceptionalism. Instead, they are formed through relational positioning, mutual referencing, and uneven participation in global networks of AI production and governance (Chung, 2025). The notion of *co-constitution* does not assume equal footing among actors. Rather, it highlights how asymmetries and inequalities are continuously produced, negotiated, and contested within dynamic regional and global relations.

This realization led me to revisit earlier work in East Asian media and cultural studies to examine how spatio-temporal entanglement has been problematized. Cultural studies scholar Kit-wai Ma (2012) proposed the idea of compressed modernity to theorize inter-city sociocultural formations in South China, challenging binary models of Western/Eastern modernity and highlighting the macro-temporal politics of cultural formation. Similarly, concepts such as intra-Asian cultural flow (Fung, 2007), transnational proximity (Jin, 2023) and contra-cultural flow (Wu, 2013) highlight both the shared sociocultural experiences of late-capitalist temporal regime and the uneven, contradictory circulation of media content and form across borders. These frameworks offer tools for understanding how cultural and technological imaginaries manifest in dynamic and non-linear ways. They support a methodological shift toward re-regionalization (Davis and Xiao, 2021) and trans-localism, both of which prioritize inter-city and inter-referential relations over methodological nationalism. This orientation makes it possible to conceptualize AI not only as a national or global project, but as contested terrain embedded in overlapping and uneven networks of relations.

Tangle, then, is both a conceptual orientation and a methodological proposition. It calls attention to the dense and uneven spatio-temporal entanglements of infrastructure, discourse, and practice that shape AI innovation in East Asia. It encourages us to transcend binary models of diffusion or transfer, whether North to South, West to East, or in reverse, and disrupts simplistic narratives of regional exceptionalism by highlighting how AI is continuously reshaped through trans-local circulations and spatial-temporal contingencies. Methodologically, *tangle* emphasizes the importance of studying both inter-referencing and regional differentiation, as well as the multiple positionings that actors and institutions occupy within global networks of technological development. From this vantage point, I began to wrestle with another puzzle in my research: What does it mean to make AI innovation (im)possible across different contexts?

### Transplant: situated adaptation and “renovation” of AI care

To understand how AI innovation takes shape not only through top-down imaginaries but also through grounded adaptation to everyday life, I extended my empirical research to the processes of designing and implementing AI-driven care technologies in Hong Kong. Building upon feminist thought on care (Fisher and Tronto, 1990), I define care technologies as interactive media, technical systems, and connective infrastructures that assist, communicate, and perform reproductive labor to make life possible, ranging from eldercare robotics to AI-enabled mental health apps.

Initially, I approached this research using translation as a sensitizing concept. In STS, translation refers to how actors modify, displace, and negotiate contradictory interests by relating disparate “things” and creating convergences to stabilize sociotechnical alignments (Callon, 1984; Latour, 2005; Sideri and van Dijk, 2025). As my fieldwork on AI mental wellness projects in Hong Kong unfolded, I found the concept of *transplantation* to be more analytically generative. Unlike translation, which often emphasizes alignment and convergence, transplantation evokes an ecological metaphor and draws attention to processes of rerooting, frictional adaptation, and partial failure. A transplanted technology, like a grafted plant, must contend with new configurations of infrastructure, culture, and institutional constraints. Transplantation also reveals the possibility of decay. Not all sociotechnical systems survive or operate seamlessly. Failure is not an exception but part of the organic process through which technologies are relocated and reshaped.

Two contrasting vignettes from my ongoing fieldwork illustrate the dynamics of transplantation.(^1^) The first involves a collaborative project between public and private sectors to develop a Cantonese-language AI chatbot for individuals experiencing emotional distress. Although the chatbot was built on globally (Western-centric) standardized therapeutic models and open-source infrastructure, localization required more than interface-level translation. Developers and researchers needed to address culturally embedded and institutionally bounded issues, for example: How should the chatbot interpret indirect or euphemistic expressions of distress, which are common in Cantonese communication? What tone and pacing would foster trust and safety without breaching norms of emotional restraint and relational distance? I found that these were not simply UX design concerns, but forms of *epistemic work*, which I refer to the human and non-human labor involved in categorizing, annotating, verifying, and interpreting data within specific institutional and cultural contexts of care. Making mental states legible and intervenable, therefore, required multiple layers of epistemic work configured not only by psychotherapeutic models and algorithmic logic, but also by assumptions about communicative norms that privilege standardizable, middle-class emotional styles (Illouz, 2008), while marginalizing subaltern or culturally divergent understandings of distress. As a result, lived experiences that deviate from dominant norms are often erased in the pursuit of technological objectivity (Hong, 2020).

The second vignette offers another perspective on transplantation. In one interview, a social worker recalled attempts to implement AI-powered eldercare robots, developed in Europe and Japan, within residential facilities and social service centers in Hong Kong. These robots faced challenges in recognizing Cantonese-accented speech and were ill-suited to the limited space typical of local housing. Rather than discarding the technology, possibly due to administrative expectations or funding commitments, care workers devised practical solutions. They rearranged interiors, created scripted prompts, and interpreted technical glitches as endearing traits that could help foster emotional connection between humans and machines. In this situation, innovation involves not seamless adoption but ongoing *renovation*. Through repeated work of repair and improvisation, the very idea of “smartness” and “care” is redefined in these circumstances. Some infrastructural barriers, however, could not be addressed. As another interlocutor observed, “a staircase won’t widen.” This succinct remark underscored how an urban built environment imposes firm limits on the flexibility of technological systems. In this case, failure did not occur as a singular disruption but persisted as a structural condition. The continued operation of transplanted systems depended on fragile, partial, and labor-intensive forms of adjustment and repair.

These encounters with adaptation and renovation led me to reconsider how media scholars conceptualize the localization of technology. What stood out was not only how tools are modified and altered, but how ideas surrounding AI care are re-rooted through lived experience. While recent critical algorithm scholarship has emphasized, in both empirical and methodological senses, the ways users interpret and relate themselves to automated media (Kaun and Männiste, 2025; Ruckenstein, 2023), I found particular resonance in the earlier scholarship of Japanese media anthropologist Takahashi (2007), whose methodological proposal for de-westernizing media studies stemmed from her ethnographic research with Japanese audiences. Takahashi outlines three key procedures: reconstructing local emic concepts to bring them into broader comparative dialogues; recontextualizing Western emic concepts that are often treated as universally applicable; and developing an integrated, internationalized framework that reflects the layered complexity of media engagement in the context of globalization. In a similar vein, Jiyeon Kang (2021) offers an analysis of post–Cold War South Korea that illustrates how transplantation involves conceptual reorganization rather than simple adoption. In her account, the concept became a productive vocabulary for envisioning a post-authoritarian society only through its reworking in relation to local historical struggles and broader global shifts, including authoritarian shadows of the past and the illiberal turns of the contemporary Global North. In my own research, these procedures and conceptual reorganization unpacked through dialogic encounters with engineers, care workers, and local researchers who were all reimagining and redefining what AI could and should signify in context. In this sense, recontextualization was a continuous process that unsettled categories and destabilized the theoretical premises of AI critique.

Transplantation, as an analytic lens, enriches critical AI studies by foregrounding the complexity and incompleteness of sociotechnical circulation. It complicates idealized notions of localization that presume smooth cultural and technical compatibility, drawing attention instead to frictions, improvisations, and occasional breakdowns that shape the institutional and cultural life of AI systems. This perspective invites critical AI scholars to examine how technologies take root in specific ecologies, and how ordinary users, professional practitioners, and infrastructures collectively sustain adaptation through ongoing acts of negotiation and maintenance. Much like non-native plants adapting to unfamiliar soil, transplanted technologies survive, and sometimes thrive, only through situated processes of ecological adjustment.

### Transmute: philosophical reorientations in AI design

While transplantation highlights situated adaptation in sociotechnical, institutional and cultural contexts, transmutation points toward a deeper reorientation of the philosophical coordinates through which AI is imagined, enacted, and made meaningful. This shift surfaced intermittently throughout my research, through unexpected encounters with AI design thinking in which intelligence, emotion, and care were not only socio-technically localized but conceptually reconfigured. These troubling moments direct me to the question of philosophical plurality: what if AI systems are not only built differently, but *imbued with meaning differently*? This problematic has long underpinned East Asian media and communication scholarship, particularly within the tradition of de-westernizing media studies. Drawing from Buddhist, Confucian, and other non-Western traditions, scholars working within this living tradition have challenged liberal assumptions about rationality, affect, and ethics embedded in dominant Northern theories of communication (Craig and Xiong, 2022). These contributions offer epistemological foundation and regional values for rethinking categories such as relation, agency, and morality in sociotechnical systems.

I encountered this philosophical reorientation most vividly while preparing for a field visit to the Gerontech and Innovation Expo cum Summit (GIES), Hong Kong’s largest public showcase of eldercare technology since 2017.(^2^) Promotional materials frequently referenced Japanese companion robots, prompting memories of familiar cultural icons such as Astro Boy and Doraemon. In Japan, robotic engineering is deeply influenced by cultural and philosophical traditions like Shinto and Buddhism, where robots are often envisioned not as tools that mimic human behavior or simulate human emotions, but as living moral agents that coexist with humans (Robertson, 2018). Building on this perspective, Daniel White and Hirofumi White and Katsuno (2025) examine how Buddhist ethics inform the design of Japanese robotics. In contrast to Western affective computing, which treats emotion as quantifiable data to be labeled and optimized, two anthropologists analyze robotic machines, such as *Mindar* and AI mindfulness coaches, that embody and elicit ethical states including compassion. They argue that these machines do not only simulate emotion but cultivate it, offering a vision of emotion that is relationally and spiritually grounded.

As I carried these encounters forward, I was drawn to philosopher Hui’s (2021) call to rethink *technodiversity* as a speculative response to the philosophical homogenization of technology. Hui challenges the assumption of a universal model of intelligence, arguing that different cosmologies give rise to plural conceptions of thought, relationality, and technical form, including fundamentally divergent definitions of intelligence itself. Drawing on New Confucian philosopher Mou Zongsan, Hui discusses the notion of intellectual intuition in Chinese thought: a mode of knowing that stands in contrast to the analytic reasoning typically privileged in dominant, calculative models of AI. The analytic of transmutation, in this sense, contributes to critical AI studies by expanding its scope beyond critiques of bias, extraction, or automation, and by redirecting attention to the philosophical foundations that demarcate how intelligence and even life are stabilized and legitimized. This reorientation invites us to take seriously the plural ontologies, epistemologies, and what Hui (2020) refers to as *cosmotechnics* that inform non-universalist approaches to technological development. Critique is no longer positioned as the fixed standpoint of a handful of paradigms, but is instead opened to transformation through dialogue and mutual flourishing with divergent philosophical traditions that shift the axis of critical examination.

## Traveling AI otherwise

This commentary is guided by a broader question: How might we understand *critical AI studies* itself as a traveling formation, and how can a traveling approach reorient critique *otherwise*?

In this concluding section, I return to ongoing projects of de-westernizing media and communication studies, a critical intervention against the globalization of Western-hegemonic academia and its cultural imperialism. De-westernization is both an intellectual and political movement, advocating for fundamental shifts in research agendas, theoretical frameworks, academic cultures, and knowledge infrastructures that center diverse epistemologies and ontologies (Waisbord, 2022; Waisbord and Mellado, 2014). As Albuquerque (2021) argues, global knowledge infrastructures continue to concentrate authority in a few elite academic nodes. Even efforts to provincialize the West often result in critique gaining traction only when rendered legible to dominant circuits of knowledge production. Qiu (2025) warns against *atheoretical comparativism* in global communication studies, where non-Western cases become empirical supplements rather than theoretical interlocutors. Jin (2021) similarly calls for theoretical re-foundation through Asian digital cultures, advocating for conditions that support collaborative projects, spreadability of locally originated work, interdisciplinarity, historical process, and the creation of local paradigms within the global context.

In this spirit, *traveling AI* is not a neutral process of theoretical diffusion, but a *reflexive praxis of in-betweenness.* As both a conceptual and methodological strategy, it describes how critical vocabularies are reworked and reinvented across sociotechnical, spatial, institutional, and cultural contexts. The three conceptual vectors developed in this commentary—tangle, transplant, and transmute—respond to this agenda by offering tools that support a more grounded, pluralistic, and globally attuned critical AI scholarship.

Table 1 outlines these orientations and their contributions to critical AI studies. Tangle illuminates relational co-constitution in spatial, temporal, and ideological terms. Transplant emphasizes frictional adaptation and “ecological conditions” in cultural and institutional contexts. Transmute opens space for philosophical reconfiguration grounded in non-Western ontologies. Together, they reconfigure critique away from universalist abstractions and toward situated epistemic engagements that move with, and through, layered differences. While this reflection is grounded in an East Asian standpoint, it does not claim regional coherence or theoretical exceptionalism. East Asia is a heterogeneous site, let alone “Asia,” among many where the epistemic foundations of critical AI studies are being actively reimagined.

**Table 1.** A summary of three conceptual orientations.

Vector	Epistemic orientation	Analytical contribution to critical AI studies
*Tangle*	Relational co-constitution across spatial, temporal and ideological contexts	Illuminates how AI imaginaries and actual innovation are shaped through inter-referencing, geopolitical relationality, and differential temporalities beyond national or linear logics.
*Transplant*	Situated adaptation through frictions, infrastructural conditions and even failure in new cultural and institutional ecologies	Highlights how AI systems are rerooted via improvisation, repair, and layered epistemic work, emphasizing adaptation as uneven and contested.
*Transmute*	Philosophical reconfiguration and pluralism of foundational concepts in AI design	Opens critique to non-Western ontologies and ethical traditions, expanding how cultural ideas of, for example, intelligence, emotion and even life can be imagined and enacted.

To travel otherwise is to travel with trouble, demanding attentiveness to what remains, what is remade, or what is left unresolved. For critical AI studies to thrive beyond its dominant formations, it must stay open to the frictions and potentialities that accompany critiques as they move, not seamlessly, but generatively.

## Notes

1. The findings on transplantation and epistemic work were presented on September 3, 2025, at the Annual Meeting of the Society for Social Studies of Science (4 seconds) in Seattle, in a talk entitled ‘(De)Coding the Mind: Epistemic Work of an AI-driven Mental Health App in Hong Kong.’

2. At the time of writing, the Gerontech and Innovation Expo cum Summit I planned to visit is scheduled for November 2025.
